# Implementation and Effects of Risk-Dependent Obstetric Care in the Netherlands (Expect Study II): Protocol for an Impact Study

**DOI:** 10.2196/10066

**Published:** 2018-05-04

**Authors:** Pim van Montfort, Jessica PPM Willemse, Carmen D Dirksen, Ivo MA van Dooren, Linda JE Meertens, Marc EA Spaanderman, Maartje Zelis, Iris M Zwaan, Hubertina CJ Scheepers, Luc JM Smits

**Affiliations:** ^1^ Care and Public Health Research Institute Department of Epidemiology Maastricht University Maastricht Netherlands; ^2^ Care and Public Health Research Institute Department of Clinical Epidemiology and Medical Technology Assessment Maastricht University Medical Centre Maastricht Netherlands; ^3^ Department of Obstetrics and Gynecology Sint Jans Gasthuis Weert Weert Netherlands; ^4^ School for Oncology and Developmental Biology Department of Obstetrics and Gynecology Maastricht University Medical Centre Maastricht Netherlands; ^5^ Department of Obstetrics and Gynecology Zuyderland Medical Centre Heerlen Netherlands; ^6^ Department of Obstetrics and Gynecology Laurentius Hospital Roermond Netherlands

**Keywords:** cohort study, pregnancy, decision support techniques, prediction model, implementation, prevention, pre-eclampsia, gestational diabetes mellitus

## Abstract

**Background:**

Recently, validated risk models predicting adverse obstetric outcomes combined with risk-dependent care paths have been made available for early antenatal care in the southeastern part of the Netherlands. This study will evaluate implementation progress and impact of the new approach in obstetric care.

**Objective:**

The objective of this paper is to describe the design of a study evaluating the impact of implementing risk-dependent care. Validated first-trimester prediction models are embedded in daily clinical practice and combined with risk-dependent obstetric care paths.

**Methods:**

A multicenter prospective cohort study consisting of women who receive risk-dependent care is being performed from April 2017 to April 2018 (Expect Study II). Obstetric risk profiles will be calculated using a Web-based tool, the Expect prediction tool. The primary outcomes are the adherence of health care professionals and compliance of women. Secondary outcomes are patient satisfaction and cost-effectiveness. Outcome measures will be established using Web-based questionnaires. The secondary outcomes of the risk-dependent care cohort (Expect II) will be compared with the outcomes of a similar prospective cohort (Expect I). Women of this similar cohort received former care-as-usual and were prospectively included between July 1, 2013 and December 31, 2015 (Expect I).

**Results:**

Currently, women are being recruited for the Expect Study II, and a total of 300 women are enrolled.

**Conclusions:**

This study will provide information about the implementation and impact of a new approach in obstetric care using prediction models and risk-dependent obstetric care paths.

**Trial Registration:**

Netherlands Trial Register NTR4143; http://www.trialregister.nl/trialreg/admin/rctview.asp?TC=4143 (Archived by WebCite at http://www.webcitation.org/6t8ijtpd9)

## Introduction

Perinatal mortality plays a pivotal role in the quality assessment of perinatal care [1]. In developed countries the main causes of perinatal mortality are small-for-gestational-age infancy (SGA), preterm birth (PTB), and asphyxia [2,3]. Pre-eclampsia (PE) is an important cause for both SGA and induced PTB [4]. Risks of asphyxia and birth injuries are increased among infants that are large-for-gestational-age (LGA) [5], which in turn is strongly associated with gestational diabetes mellitus (GDM) [6]. Thus, PE, GDM, PTB, SGA, and LGA are all directly or indirectly related to perinatal mortality.

A number of interventions have shown to be effective in the prevention of adverse pregnancy outcomes, such as calcium suppletion and low-dose aspirin treatment in case of PE [7-9], adequate management of GDM [5,10,11], and progesterone administration in women at risk of spontanous PTB [12]. Besides calcium supplementation, most of these interventions are not suitable for all pregnant women, because of either possible adverse effects, patient burden, or costs. Early prediction of obstetric risks may therefore help health care professionals in designing intervention strategies based on women’s individual risks.

Recently, we performed an external validation study of first trimester prediction models predicting the risk of PE, GDM, PTB, SGA and LGA (the Expect Study I) [13,14]. The Expect Study I identified clinically useful prediction models for PE and GDM. The Limburg Obstetric Consortium (LOC), midwives and gynecologists of the southeastern part of the Netherlands developed care pathways, for example, basic antenatal care for women at low risk and additional risk-dependent care for women with elevated risks of PE, GDM, PTB, SGA, or LGA. The LOC agreed to implement the risk models predicting PE and GDM, in order to identify women at increased risk of these outcomes, and to offer these women risk-dependent care.

The current protocol describes the design of a multicenter prospective cohort study (Expect Study II) evaluating the implementation progress of using these prediction models combined with tailored care paths for PE and GDM.

The primary aims of the Expect Study II are to measure adherence to the new risk-dependent care guidelines by health care professionals and compliance of pregnant women who received recommendations. The secondary aims are to evaluate its impact upon patient satisfaction and cost-effectiveness. Secondary aims will be studied by comparing these outcomes of the Expect II cohort with the Expect I cohort.

## Methods

### Study Design and Recruitment

In April 2017, the Expect prediction tool, was introduced. The Expect prediction tool was developed to enable individual risk assessment during early pregnancy regarding the risks of PE, GDM, PTB, SGA, and LGA. Validated models selected by the LOC to predict PE and GDM have been incorporated into this tool (unpublished study submitted by Meertens et al, January 2018). Risk assessment of spontaneous PTB, SGA and LGA is achieved using the revised LOC guidelines [15]. For nulliparous women, the prediction tool comprises 14 variables concerning anthropometric data, relevant medical history, and family history. For multiparous women the tool enquires 6 more variables, all concerning the women’s obstetric history.

The Expect prediction tool is a Web-based form which calculates the estimated risk profiles. This tool was made available for health care professionals via the Expect study website for implementation in daily obstetric care. Besides the estimated risks of adverse pregnancy outcomes, the tool provides recommendations for tailored antenatal care based on personalized risks (ie, risk-dependent care). In addition, patient information brochures relevant to the patient’s risk profile will be automatically generated. The health care professionals can use this tool during one of the pregnant woman’s antenatal visits before 16 weeks of pregnancy. Using a shared decision approach, the appropriate risk-dependent care path with corresponding preventive measures and check-ups will be selected.

In order to implement risk-dependent care successfully, midwives and gynecologists are encouraged to use the Expect prediction tool by representatives of the LOC. The Expect prediction tool is introduced by email to all obstetric health care professionals in the region. Furthermore, oral presentations will be given at every hospital and at local midwifery meetings. Additionally, the hospitals and midwifery practices are contacted regularly by phone and in person to evaluate the Expect prediction tool.

The midwives and gynecologists play a central role in enrolling pregnant women into the Expect Study II, by asking women whether they are interested in receiving further information about participating in the Expect Study II. Almost every pregnant woman is eligible for our study. The exclusion criteria are (1) maternal age <18 years, (2) documented multiple pregnancy, and (3) ≥16 weeks of gestation at intake. The eligibility criteria are identical to those of the Expect Study I cohort [13]. Eligible women agreeing to participate are asked to give informed consent and to complete 4 Web-based surveys at enrolment, 24 weeks and 34 weeks of gestation, and 6 weeks after due date.

A personal link to the first online survey will be sent immediately after enrolment. If the survey was not accessed or incomplete, 2 automatic reminders will be sent by email at 3-day intervals for surveys one to three and at 6-day intervals for the postpartum survey. In case of non-response, women will be contacted by phone (provided that a correct phone number is available). If women report PTB at the beginning of survey two or three, they will automatically be redirected to the postpartum survey.

The medical ethical committee of Maastricht University Medical Centre evaluated the study protocol and declared that the study did not fall within the scope of the Dutch Medical Research Involving Human Subjects Act (WMO; METC-17-4-057).

### Tailored-Care Paths

The LOC consists of midwives (n=9), gynecologists (n=9), professionals in maternity care (n=2), researchers (n=3), and an independent chairman. They meet four to five times annually and represent the University medical school, midwifery academy, all hospitals, and roughly 80% (n=90) of the midwives of the province. The midwives and gynecologists of the LOC revised the content of obstetric care. We will briefly describe the most important changes regarding antenatal care compared to former care-as-usual which has been observed during Expect Study I. All women will receive basic antenatal care. In the new tailored care paths, recommendations about calcium and vitamin D supplementation are emphasized for all women and an additional ultrasound for fetal growth assessment at 32 weeks of pregnancy is introduced as part of basic antenatal care.

An overview of the care pathways is provided in Table 1. Additional risk-dependent care for women with a mildly elevated risk of PE comprises the recommendation of preventive aspirin treatment, 80-100 mg aspirin daily from 12 weeks up to 36 weeks of pregnancy. Obstetric care for women with a substantial risk of PE additionally comprises of extended blood tests, blood pressure measurements every 2 weeks from 14 weeks up to 40 weeks of gestation, and 2 additional ultrasounds for fetal growth measurements.

Women with a history of GDM are advised to have an oral glucose tolerance test (OGTT) at 16 and 26 weeks of pregnancy. Women with a mildly elevated risk are advised to have an OGTT at 24 weeks of pregnancy. Furthermore, in both cases, women will receive two additional ultrasounds for fetal growth measurements in addition to basic antenatal care.

### Outcome Measures and Measurement

The primary outcomes are health care professionals’ adherence to key recommendations and compliance of the women involved in the study. Adherence is defined as the proportion of women that actually received the key recommendations they should have received from their health care professional according to the LOC guidelines. Adherence will be analyzed regarding recommendations of adequate vitamin D (yes or no) and calcium intake (yes or no) for all women, preventive aspirin treatment (yes or no) for women with elevated PE risks, and OGTT (yes or no) for women with elevated GDM risks.

Compliance is defined as the proportion of women whom comply with the LOC recommendations they have received (yes, no or partially). Compliance will be analyzed regarding: adequate vitamin D (10 microgram per day) and calcium (1,000 milligram per day) intake, preventive aspirin treatment, and OGTT.

The secondary outcomes are patient satisfaction and cost-effectiveness. These secondary outcomes of Expect Study II will be compared to the outcomes of Expect Study I.

Patient satisfaction will be measured by validated patient satisfaction questionnaires. The Patient Satisfaction Questionnaire Short Form will be incorporated in antepartum surveys two and three. In the postpartum survey, patient satisfaction will be assessed by the Pregnancy and Childbirth Questionnaire (PCQ) [16]. The PCQ is validated for Dutch women who recently gave birth and addresses three topics: women’s satisfaction with the health care professional during pregnancy, health education, and satisfaction with the health care professional during labor. Furthermore, Truijens et al showed the PCQ is sensitive to pick up effects regarding patient satisfaction due to simulation-based obstetric team training [17].

In order to perform cost-effectiveness calculations, we will calculate two incremental cost-effectiveness ratios (ICERs). The first ICER expresses the health care costs per one neonatal composite outcome prevented. The neonatal composite outcome is defined as perinatal death within seven days after birth, asphyxia (Apgar score <7 after 5 minutes), admission to a neonatal intensive care unit within 28 days after birth, SGA (birthweight <2.3 weight percentile), and very preterm birth (birth before 32 completed weeks of pregnancy) [13]. The second ICER will express the health care cost per one maternal gained Quality Adjusted Life Year (QALY).

### Data Collection

For the primary outcomes, we will use the data collected for the Expect Study II. For the secondary outcomes, when comparing the effects of risk-dependent care with former care-as-usual, the outcomes of the Expect Study II will be compared with the outcomes of the Expect Study I. For this reason, the survey intervals and the questions regarding the secondary outcomes are similar between the two studies.

**Figure 1:**
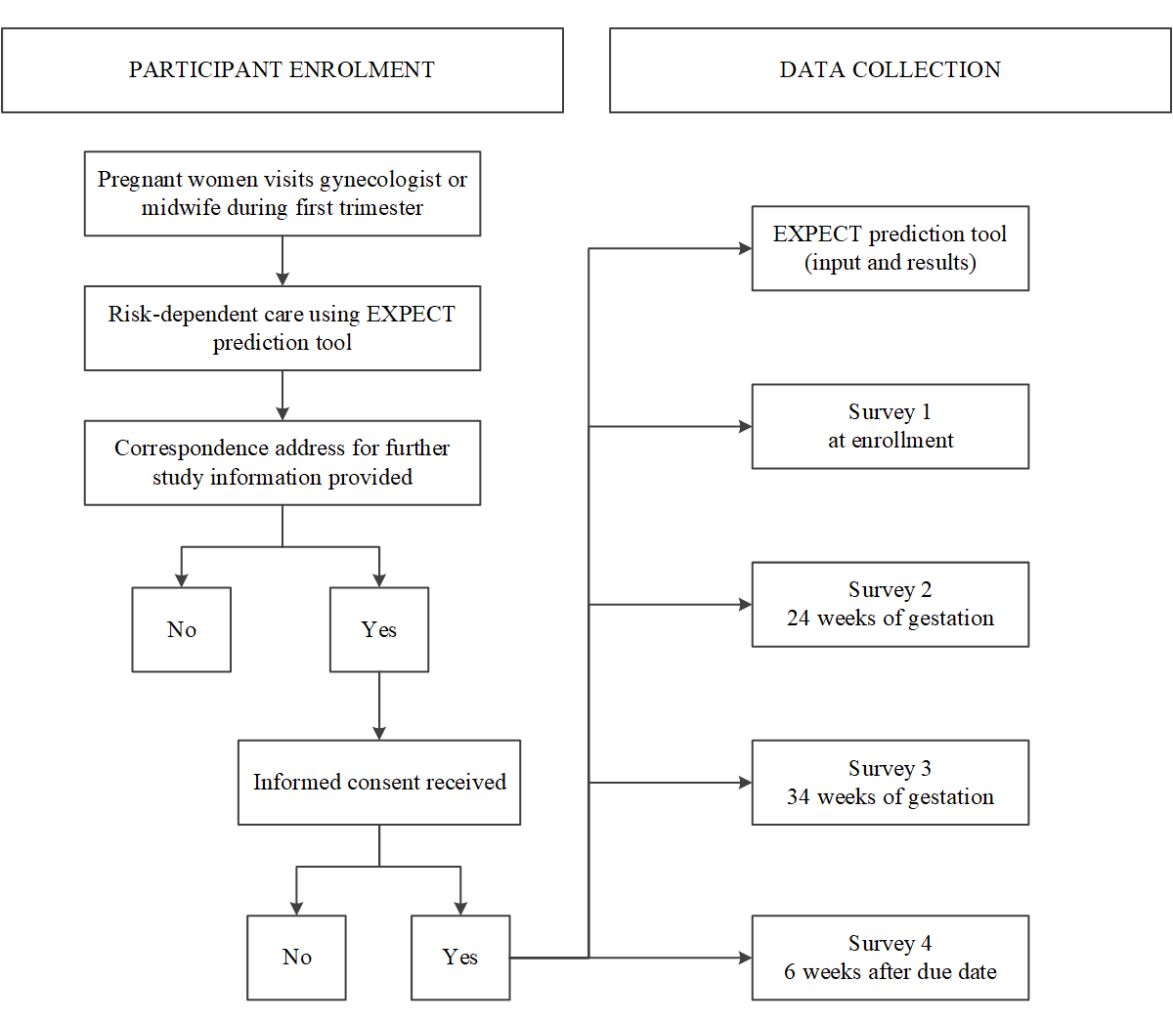
Flowchart of participant enrolment and data collection of the Expect Study II. Whether or not a woman participates to the Expect Study II does not affect the health care women receive during their pregnancy.

In the Expect Study II, data will be collected using the Expect prediction tool, comprising women’s personal risk profile, and Web-based patient surveys. A structured overview of patient enrolment and data collection for the Expect Study II is shown in Figure 1.

The first survey addresses the following topics: (1) recommendations and information given by health care professionals, (2) women’s intention to comply with these recommendations, (3) dietary intake of calcium and vitamin D and sunlight exposure, and (4) vitamin and mineral supplement usage.

The second and third surveys are comparable to each other and will address the following topics: (1) patient satisfaction, (2) women’s state anxiety, (3) maternal quality of life, (4) changes in vitamin and mineral supplement usage, and (5) health care resource use.

**Table 1:** Overview of care pathways.

Gestational age (weeks)	Basic antenatal care for all women	Additional risk-dependent care				
		Pre-eclampsia (mildly elevated risk)	Pre-eclampsia (high risk)	Gestational diabetes mellitus	Small or large gestational age infancy	Spontaneous preterm birth
6-10	Intake and risk assessment using the Expect prediction tool and general recommendations (eg, Calcium and vitamin D intake)					
10-12	Confirmation gestational age (crown rump length ultrasound) and blood tests (eg, blood type, hemoglobin)	Low dose aspirin prophylaxis	Low dose aspirin prophylaxis and extended blood tests (eg, renal function)			Cervical length measurement
14			BP^a^ measurement			
16			BP measurement	OGTT^b^ (in case of history of GDM^c^)		Cervical length measurement and progesterone prophylaxis
18-20	Check-up (eg, BP and symphysio-fundal height measurements) and ultrasound screening for congenital abnormalities					Cervical length measurement
22			BP measurement			
24-26	Check-up		BP measurement	OGTT		Cervical length measurement
27	Additional blood tests (depending on Rhesus genotype)					
28			BP measurement and ultrasound fetal biometry	Ultrasound fetal biometry	Ultrasound fetal biometry	Cervical length measurement
30	anti-D immunoglobulin prophylaxis (depending on genotype)					
32	Check-up, blood tests (eg, hemoglobin), and ultrasound fetal biometry					Cervical length measurement
34			BP measurement			
36	Check up and ultrasound fetal biometry		Ultrasound fetal biometry		Ultrasound fetal biometry	
37			BP measurement			
38			BP measurement			
39			BP measurement			
40	Check-up and shared decision regarding induction of labor					
41-42	Check-up					

^a^BP: blood pressure.

^b^OGTT: oral glucose tolerance test according to the International Association of the Diabetes and Pregnancy Study Group's (IADPSG) criteria.

^c^GDM: gestational diabetes mellitus.

In order to document the nature and volume of health care resource used, women will be asked to record all visits to midwives, hospitals, and other care institutions. Furthermore, questions related to medication use, hospital admission, diagnostic and medical procedures, and the delivery will be asked. To minimize patient recall problems, information regarding the usage of health care resources will be requested at three intervals (surveys two, three and four) during the study period.

Survey four, the postpartum survey, addresses obstetric outcomes, compliance of health care recommendations, and the topics mentioned in survey two and three. Furthermore, this survey also contains questions regarding the health care consumption related to the neonate.

### Sample size

According to the results of the validation study (Expect I) we expect approximately 30% of women to have an elevated estimated risk of PE, the obstetric complication with the lowest incidence (unpublished study submitted by Meertens et al, January 2018). Furthermore, an adherence of 70% and a compliance of approximately 40% is expected for the recommended aspirin treatment. This will result in approximately 21% and 12% respectively of the general population having an elevated risk of PE. In order to estimate these percentages with a precision of approximately 4% the required sample size is estimated at 400 participants [18].

### Statistical Analysis

Missing values will be handled by imputation. Stochastic regression imputation with predictive mean matching as the imputation model will be used to prevent biased results based on complete case analysis only [19].

Adherence will be calculated by the proportion of women who reported to have received the LOC recommendations regarding adequate vitamin D and calcium intake, preventive aspirin treatment, and OGTT. Answers of participants will be linked to their estimated risk profile based on the Expect prediction tool.

Compliance will be analyzed by calculating the proportion of women who complied with the recommendations received from their health care professional regarding aspirin treatment, OGTT, vitamin D, and calcium intake. Vitamin D is analyzed based on supplement intake and sunlight exposure. Calcium intake is determined by calculating the daily intake from diet and supplement use. Dietary intake will be estimated using answers from a selection of questions from the Dutch National Food Frequency Questionnaire tool [20]. These questions address food products that cumulatively cover >80% of the variance in calcium intake [21]. Total intake of both nutrients will be compared with the recommended intake by the LOC (1000 milligram calcium per day and 10 microgram of vitamin D per day) in order to determine compliance to these recommendations.

The secondary outcomes, patient satisfaction and cost-effectiveness, will be analyzed by comparing Expect Study II with the outcomes of former care-as-usual (Expect Study I). Patient satisfaction scores will be analyzed using multiple linear regression.

For the economic evaluation, we will use a health care perspective according to the Dutch guidelines for cost calculations [22]. A time horizon of approximately eleven months, from onset of pregnancy up to six weeks post-partum, will be applied. Maternal quality of life will be evaluated using the Euroqol EQ-5D-3L and EuroQol Visual Analogue Scale (EQ VAS) questions which are incorporated in the surveys. The EQ-5D-3L and EQ VAS are standardized questionnaires used worldwide to assess quality of life. Maternal QALYs will be calculated using the corresponding utility scores based on the Dutch population [23,24]. All costs will be expressed as 2017 Euros and if necessary cost prices will be transformed to 2017 Euros using the Dutch Consumer Price Index [25]. Bootstrap- and standard sensitivity analyses will be performed to quantify the uncertainty regarding the cost-effectiveness outcomes.

## Results

Currently, women are being recruited for the Expect Study II and a total of 300 women are enrolled. We expect to achieve our goal of 400 participants during April 2018 and postpartum data collection will be finished by March 2019. As a result, first study results are expected in 2019.

## Discussion

This paper describes the protocol of an impact study regarding the implementation of externally validated prediction models combined with risk-based care pathways in obstetric care. Prediction models are becoming increasingly popular in medicine [26]. Although the number of prediction models being published has increased tremendously in recent years, the number of external validation studies remains small [26]. Furthermore, performances of models predicting adverse pregnancy outcomes and the efficacy of preventive interventions for these outcomes are generally documented separately [8,27,28]. Impact studies, describing the effect of using prediction models in daily practice combined with preventive interventions relevant to the estimated risk are nearly non-existent [26]. To the best of our knowledge no impact studies using prediction models in general obstetric practice have been published.

The strengths of our design are the multicenter prospective data collection and the similarity of both cohorts. Recruitment in multiple centers, hospitals and midwife clinics, improves the probability of collecting a representative sample of the obstetric population. This is essential in the Netherlands, since most pregnant women receive antenatal care by midwives at outpatient clinics [29]. Furthermore, optimal measurement of the outcomes is achieved by prospective data collection [30]. Finally, because the two cohorts are kept as similar as possible, we are able to accurately compare the former care-as-usual with the new risk-dependent care.

Some limitations of the design must also be noted. First, since the comparison of secondary outcomes of Expect II with those of Expect I is essentially a before-and-after comparison, time trends in the outcomes can theoretically influence results. In the interpretation of the results, we will take such trends into account, fro exmaple, by looking at trends in the studied outcomes from other regions in the Netherlands.

A second possible limitation of our study is that several outcomes will solely be based on participant questionnaires. Potential recall bias, however, is limited due to the prospective design and the usage of 4 questionnaires at limited intervals. Additionally, questionnaires have been shown to be a valid method of data collection regarding perinatal outcomes and medication exposure during pregnancy [31,32]. In the questionnaires we urge respondents to answer honestly and emphasize that all answers will be treated confidentially and will not influence the care provided by their obstetric health care professional. Furthermore, the additional procedures recommended in the risk-dependent care path are all subject to a shared decision-making process between woman and health care professional. As a result, we expect there is currently no taboo regarding the compliance with given recommendations.

We hypothesize that risk-dependent care results in early detection or prevention of obstetric adverse events and can thus reduce prevalence of neonatal adverse events. However, due to low prevalence rates of approximately 5%, large cohorts (approximately two times 6,800 participants) are necessary in order to achieve sufficient power to detect a reduction of at least 20% [18]. Therefore, the influence of risk-dependent care on the incidence of the neonatal composite outcome will be analyzed using registry data of the region. Moreover, to achieve the desired effects of risk-dependent care, it first needs to be implemented successfully. Thus, implementation should first lead to behavioral changes for both health care professionals and pregnant women.

## Appendix

Multimedia Appendix 1 Peer-reviewer report.

## Abbreviations

BP: blood pressure
EQ VAS: EuroQol Visual Analogue Scale
GDM: gestational diabetes mellitus
IADPSG: International Association of the Diabetes and Pregnancy Study Group
ICER: incremental cost-effectiveness ratio
LGA: large-for-gestational-age
LOC: Limburg Obstetric Consortium
OGTT: oral glucose tolerance tes
PCQ: Pregnancy and Childbirth Questionnaire
PE: pre-eclampsia
PTB: preterm birth
QALY: Quality Adjusted Life Year
SGA: small-for-gestational-age infancy
